# Neurodegeneration and Unfolded-Protein Response in Mice Expressing a Membrane-Tethered Flexible Tail of PrP

**DOI:** 10.1371/journal.pone.0117412

**Published:** 2015-02-06

**Authors:** Paolo Dametto, Asvin K. K. Lakkaraju, Claire Bridel, Lukas Villiger, Tracy O’Connor, Uli S. Herrmann, Pawel Pelczar, Thomas Rülicke, Donal McHugh, Arlind Adili, Adriano Aguzzi

**Affiliations:** 1 Institute of Neuropathology, University Hospital Zurich, Zurich, Switzerland; 2 Institute of Laboratory Animal Science, University of Zürich, Zurich, Switzerland; 3 Institute of Laboratory Animal Science, University of Veterinary Medicine Vienna, Vienna, Austria; Van Andel Institute, UNITED STATES

## Abstract

The cellular prion protein (PrP^C^) consists of a flexible N-terminal tail (FT, aa 23–128) hinged to a membrane-anchored globular domain (GD, aa 129–231). Ligation of the GD with antibodies induces rapid neurodegeneration, which is prevented by deletion or functional inactivation of the FT. Therefore, the FT is an allosteric effector of neurotoxicity. To explore its mechanism of action, we generated transgenic mice expressing the FT fused to a GPI anchor, but lacking the GD (PrP_Δ141–225_, or “FTgpi”). Here we report that FTgpi mice develop a progressive, inexorably lethal neurodegeneration morphologically and biochemically similar to that triggered by anti-GD antibodies. FTgpi was mostly retained in the endoplasmic reticulum, where it triggered a conspicuous unfolded protein response specifically activating the PERK pathway leading to phosphorylation of eIF2α and upregulation of CHOP ultimately leading to neurodegeration similar to what was observed in prion infection.

## Introduction

**Fig 1:**
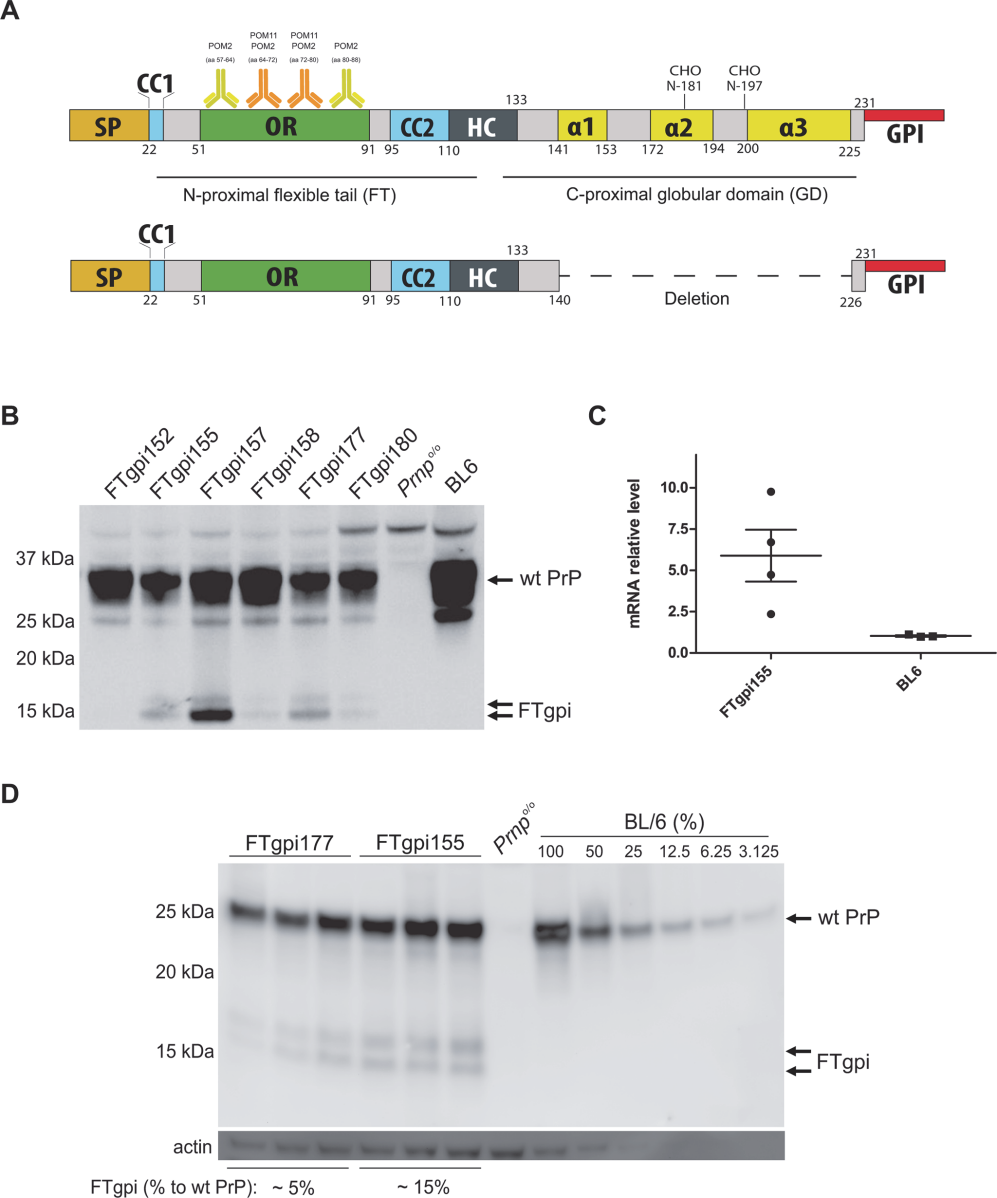
Expression of FTgpi protein. (**A**) Scheme of wild-type PrP and FTgpi utilized for the generation of the transgenic mouse. SP: signal peptide; OR: octapeptide repeats; CC2: charge cluster 2; HC: hydrophobic core; α1, α2 and α3: α-helixes; GPI: glycosylphosphatidylinositol anchor. POM2 binds the degenerate epitope QPHGGG/SW (aa 57–64, 64–72, 72–80, 80–88) whereas POM11 recognizes the epitope QPHGGSW (aa 64–72, 72–80) of the FT [16]. (**B**) Brains of FTgpi, *Prnp* ^o/o^ (here and henceforth negative control) and B6/J-Hsd1 mice (BL6, used as positive control here and henceforth) were analyzed by Western blotting using POM11 antibody. Double arrow: FTgpi protein (17 kDa and 15 kDa). The FTgpi157 founder mouse, which showed the highest expression, died shortly after birth. (**C**) Transgenic FTgpi and wt PrP transcription quantified by Real-Time PCR (RT-PCR). FTgpi mRNA was ca. 6x higher than *Prnp* mRNA. (**D**) Brain homogenates were subjected to PNGase F treatment. FTgpi155 and FTgpi177 mouse lines displayed protein levels of 15% and 5%, respectively, compared to a calibration curve with serially diluted BL6 brain (not shown). Western blots were performed using POM2.

The cellular prion protein (PrP^C^) is a GPI-anchored membrane glycoprotein whose conversion into a misfolded, aggregated conformer (PrP^Sc^) is the central event in prion diseases [1]. PrP^C^ consists of an unstructured, flexible N-terminal tail (FT, residues 23–128) hinging on a compact globular domain (GD, residues 129–231). Whereas the GD comprises three α-helices (α1, α2 and α3) and two-stranded antiparallel β-sheets [2], nuclear-magnetic resonance spectroscopy has shown that the FT is entirely unstructured [3]. The amino acid sequence of the FT is composed of the signal peptide (SP) followed by the small “charge cluster 1” (CC1), a series of octapeptide repeats (OR), the “charge cluster 2” (CC2), and the hydrophobic core (HC) – a stretch of 20 hydrophobic amino acids linking the FT with the GD (Fig. 1A). CC2 and HC together form the central domain (CD). These domains have been reported to interact with a broad range of proteins and to endow the FT with a number of potential, yet poorly understood, properties [4]. However, a unifying mechanistic framework of the function of PrP is still lacking.

Antibodies targeting the α1 and α3 helices of the GD are profoundly neurotoxic, and their toxicity is strictly dependent on an intact FT [5]. These phenomena point to an allosteric mechanism of action: conformational transitions in the GD may influence the topology of the FT relative to the plasma membrane or to other cellular constituents, and may eventually trigger a cascade of deleterious events (S1 Fig.). As predicted from this model, removal of the OR region from the FT, or pretreatment with anti-OR antibodies, abolishes the toxicity of anti-GD antibodies.

The downstream effectors of the cascade delineated above include the production of toxic superoxide and the activation of calpains, but the proximal events are unknown. In order to elucidate the nature of the latter, we have generated transgenic mice expressing the FT directly fused to a GPI anchor, yet devoid of the entire GD (PrP_Δ141–225_, henceforth termed “FTgpi”). We found that FTgpi-expressing mice developed progressive, lethal neurodegeneration. Toxicity was associated with chronic ER stress and activation of the PERK pathway, which eliminates damaged cells by apoptosis [6–9], similarly to events occurring in *bona fide* prion infections [10].

## Results

### Generation of transgenic mice and analysis of protein expression

To investigate the possible toxicity of FT *in vivo*, a redacted version of the prion protein lacking the globular domain but retaining the GPI anchor signal (PrP_Δ141–225_ or FTgpi; Fig. 1A) was cloned into the “half-genomic” pPrPHG backbone, which contains the *Prnp* gene devoid of intron #2 [11]. The resulting construct was injected into pronuclei of *Prnp* ^+/+^ mice. Potential founders were screened by tail PCR for the presence of the transgene, and six founders were identified as carriers. Three transgenic lines (FTgpi155, FTgpi157, and FTgpi177) were selected for further analysis on the basis of transgenic protein expression levels in the CNS (Fig. 1B). FTgpi was visualized as a double band at 15 kDa and 17 kDa, suggesting that FTgpi probably assumes distinct transmembrane topologies affecting cleavage of its signal peptide and/or its GPI-anchored signal.

The highest expressing founder, FTgpi157, and its offspring died before further analyses could be performed. This was the first indirect observation suggesting that FTgpi could be toxic. The two remaining lines (FTgpi155 and FTgpi177) were crossed with *Prnp* ^o/o^ mice to obtain offspring lacking PrP^C^. The transgene was inherited as an autosomal trait at Mendelian frequency (S1 Table). Transgenic mRNA levels in FTgpi155 brains were ~6-fold higher than *Prnp* mRNA in C57BL/6 mice (BL6; Fig. 1C), yet FTgpi protein levels were 15% (FTgpi155) and 5% (FTgpi177) of total PrP^C^ in BL6 brains (Fig. 1D).

### Pathological phenotypes of mice expressing FTgpi

**Fig 2:**
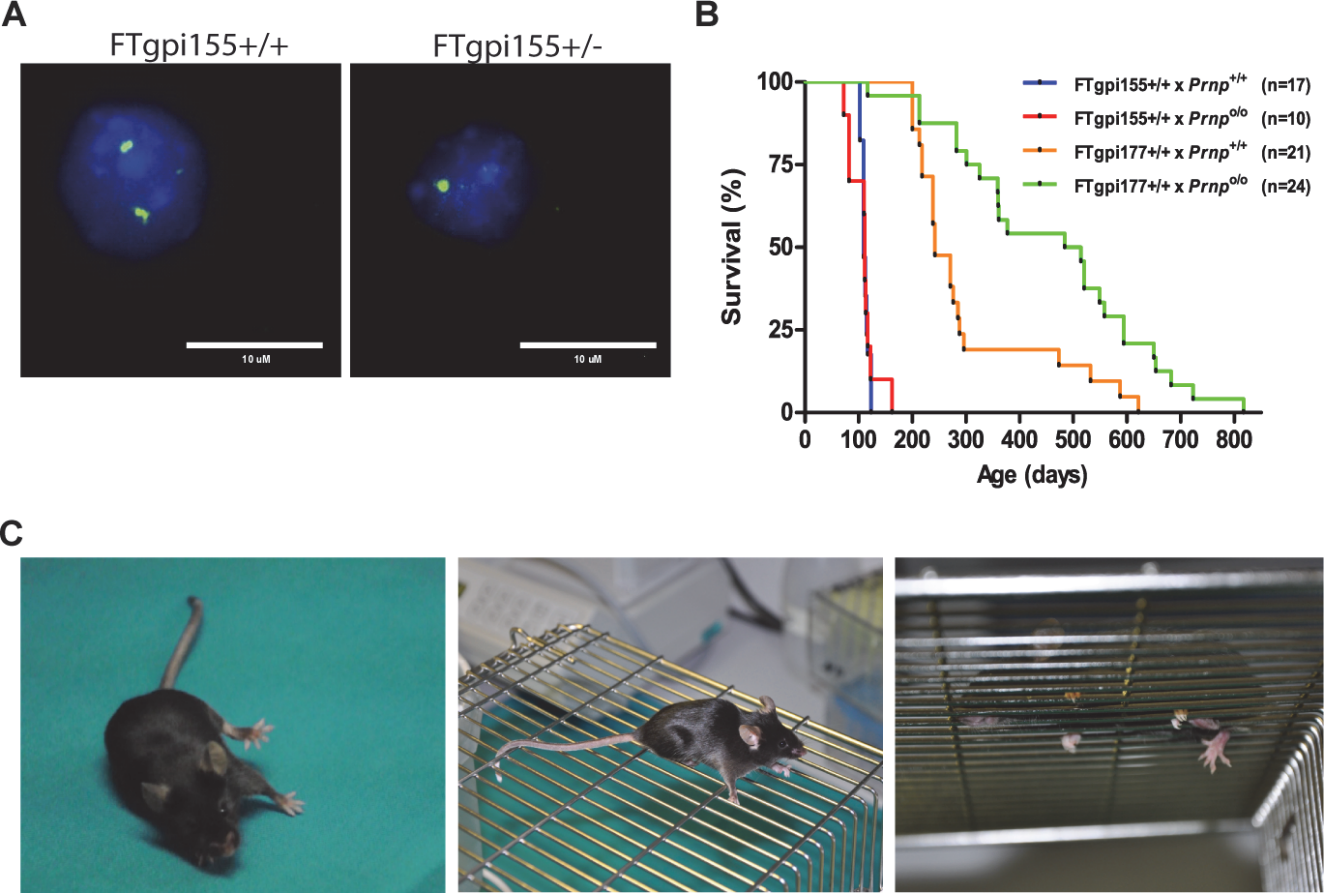
Survival of FTgpi mice. (**A**) ZyFISH results. Examples of nuclei which show either one (FTgpi155+/-) or two bright fluorescent signals (FTgpi155^+/+^), indicative of heterozygosity or homozygosity for the transgene. (**B**) Survival curves of mice expressing FTgpi in the absence (x *Prnp* ^o/o^) or presence (x *Prnp* ^+/+^) of full-length PrP. The number of mice is indicated in the legend. Higher expressor FTgpi155 *Prnp* ^+/+^ and FTgpi155 *Prnp* ^o/o^ mice died significantly earlier than FTgpi177 *Prnp* ^+/+^ (p<0.0001) and FTgpi177 *Prnp* ^o/o^ (p<0.0001) mice respectively, suggesting that toxicity is dose-dependent. FTgpi177 *Prnp* ^+/+^ mice died significantly earlier than FTgpi177 *Prnp* ^o/o^ mice (p<0.001), indicating that PrP^C^ may exacerbate the phenotype. Statistics: Log-Rank (Mantel-Cox) test. (**C**) Typical clinical phenotypes at terminal stage. Left panel: FTgpi155^+/+^ mouse showing ataxia symptoms. Central and right panel: representation of a grid test. FTgpi155^+/+^ legs repeatedly falling through the grid.

All FTgpi transgenic lines were maintained in both *Prnp* ^+/+^ and *Prnp* ^o/o^ genetic backgrounds. We used the ZyFISH technique [12] to reliably discriminate between homo- and hemizygous transgene carriers (Fig. 2A). Mice were monitored according to a previously published four-degree clinical score [13]. Both the FTgpi155 and the FTgpi177 line developed a phenotypically similar form of ataxia (S1 Video). Since each line was derived from an independent pronuclear microinjection leading to unique chromosomal integration events, we conclude that the phenotype was induced by the expression of the transgene rather than by insertional mutagenesis.

The higher-expressing lines, FTgpi155 *Prnp* ^+/+^ and FTgpi155 *Prnp* ^o/o^, developed signs of disease and died significantly earlier (median ± standard deviation: 109 ± 6.7 and 111 ± 24 days, respectively) than the lower expressing lines FTgpi177 *Prnp* ^+/+^ and FTgpi177 *Prnp* ^o/o^ (242 ± 127 and 479 ± 178 days, respectively), indicating that FTgpi toxicity was dose-dependent (Fig. 2B). Co-expression of PrP^C^ did not improve the survival of FTgpi mice (Fig. 2B). This suggests that the mechanism of toxicity is different from that of the deletion mutants, PrP_Δ94–134_ and PrP_Δ32–134_, which are dose-dependently rescued by co-expression of PrP^C^ [13,14]. On the contrary, PrP^C^ seemed to worsen the phenotype in FTgpi177 mice, as FTgpi177 *Prnp* ^*+/+*^ died significantly earlier that FTgpi177 *Prnp* ^o/o^ mice (Fig. 2B, orange and green lines). This phenomenon did not occur in FTgpi155 mice, perhaps because FTgpi expression was saturating.

The first sign of disease (~9 weeks) observed in FTgpi155 *Prnp* ^+/+^ was shivering, followed by mild limping and hind limb weakness. At later stages (~12 weeks), mice tended to fall sideways while walking in the cage (Fig. 2C, left panel). In addition, mice were placed on a metal grid and monitored for 5 min to assess their walking proficiency (cage grid test). At later stages (~14 weeks), tests resulted always positive, with both legs consistently falling through the grid (Fig. 2C, central and right panels; S2 Video). No paralysis was observed.

### FTgpi induces cerebellar degeneration

**Fig 3:**
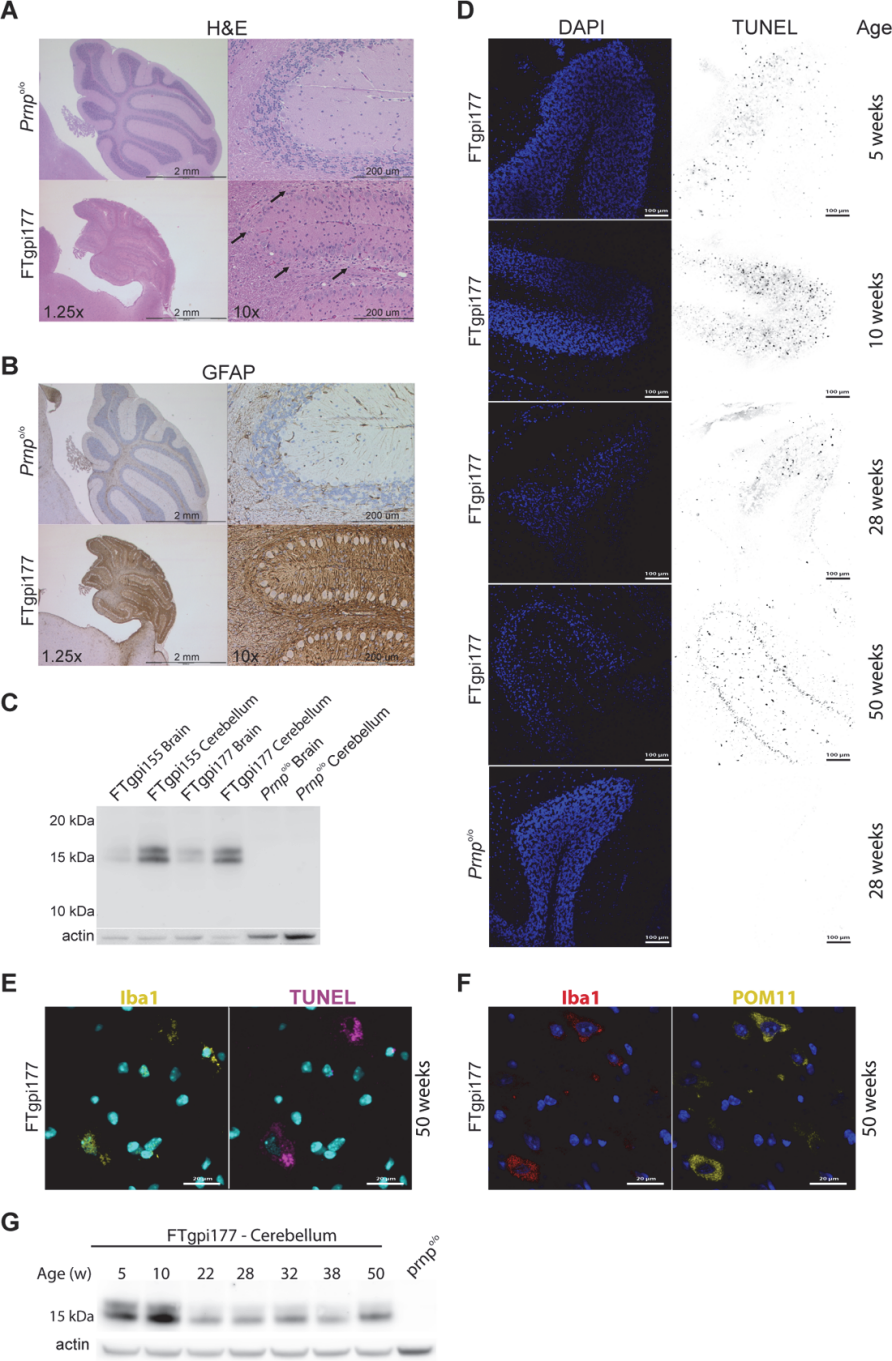
Cerebellar pathology in FTgpi mice. All histological investigations show FTgpi177 *Prnp* ^o/o^ mice; age-matched *Prnp* ^o/o^ mice were used as controls. (**A**) H&E staining showing severe atrophy of the cerebellum and conspicuous loss of CGNs in FTgpi177 mice (right panels, arrows) compared to controls (left panels). (**B**) GFAP-stained cerebellum showing widespread astrogliosis in FTgpi177 mice. (**C**) Both FTgpi155 and FTgpi177 showed increased FTgpi expression in the cerebellum. Western blot was decorated with POM2 antibody. (**D**) Cerebellar frozen sections were stained with DAPI (blue) and TUNEL (red) to reveal nuclei and fragmented DNA, respectively. A gradual loss of CGNs is evident in the granular layer. Numerous TUNEL^+^ cells were detected at 10 weeks in FTgpi mice. Frozen sections of age-matched *Prnp* ^o/o^ controls were stained at all respective time points (shown at 28 weeks) and did not reveal any TUNEL^+^ cells. (**E**) Cerebellar frozen sections were stained with DAPI (blue), Iba1 (yellow) and TUNEL (purple) to reveal nuclei, fragmented DNA, and microglia respectively. TUNEL signal localized to the cytoplasm of microglia. (**F**) Cerebellar frozen sections were stained with DAPI (blue), Iba1 (red) and POM11 (yellow) to reveal nuclei, microglia, and FTgpi. A strong FTgpi signal was detected in the cytoplasm of microglia. (**G**) Western blot using POM11 showed FTgpi protein expression decreasing over time in the cerebellum, possibly as a consequence of the progressively reduced number of neurons.

Histological examination of clinically affected FTgpi mice revealed conspicuous pathological changes in the cerebellum. All FTgpi lines displayed a similar phenotype consisting of progressive loss of hemispheric cerebellar granule neurons (CGNs; Fig. 3A, arrows), substantial thinning of the granule cell layer, widespread atrophy (Fig. 3A), and intense astrocytosis (Fig. 3B). Obvious degeneration was absent from the rest of the brain, possibly due to the lower expression level of FTgpi (Fig. 3C). Other organs were unaffected (S2 Fig.) but they also failed to express detectable FTgpi (S2 Fig.).

Terminal deoxynucleotidyl transferase dUTP nick end labeling (TUNEL) detects DNA fragmentation resulting from apoptosis or other forms of cell death. CGNs of FTgpi177 mice showed nuclear TUNEL signals already at 5 weeks of age, which became widespread by 10 weeks (Fig. 3D). Strikingly, mice did not show any symptoms at this stage. At 28 weeks the signal was reduced, probably due to the removal of dead neurons by microglia. At 50 weeks TUNEL^+^ cells shifted from the granular layer to the molecular layer and white matter (Fig. 3D), and the TUNEL signal became localized to the cytoplasm of Iba1^+^ cells (Fig. 3E), suggesting neuronophagia by microglia. Accordingly, microglia showed a robust cytoplasmic FTgpi signal (Fig. 3F). DAPI staining confirmed the progressive loss of CGNs in the cerebellum (Fig. 3D), which was further reflected in progressive decrease of FTgpi expression over time (Fig. 3G).

### FTgpi is retained in the ER

The biogenesis of FTgpi was studied by inserting the wild-type PrP^C^ and FTgpi reading frames into the expression plasmid pBMN, which was used to generate retroviral particles using the Phoenix Retrovirus Expression System (Orbigen). We infected HPL cells lacking PrP^C^ [15], and created stably-transfected cell lines expressing either FTgpi or PrP^C^. FTgpi lacks the two N-glycosylation sites that are located in the GD, and was expressed by HPL cells as an unglycosylated 15 kDa protein (S3 Fig.). To monitor if the FTgpi is indeed GPI anchored, we performed partitioning of proteins using the detergent, Triton X-114. As with WT PrP^C^, a significant fraction of FTgpi protein partitioned into the pellet fraction (S3 Fig.) suggesting that it was anchored to lipid membranes. To further confirm the modification, we performed a PI-PLC digestion on the FTgpi in the whole cell lysate and observed a faster migrating band, confirming the cleavage of the gpi anchor (S3 Fig.).

**Fig 4:**
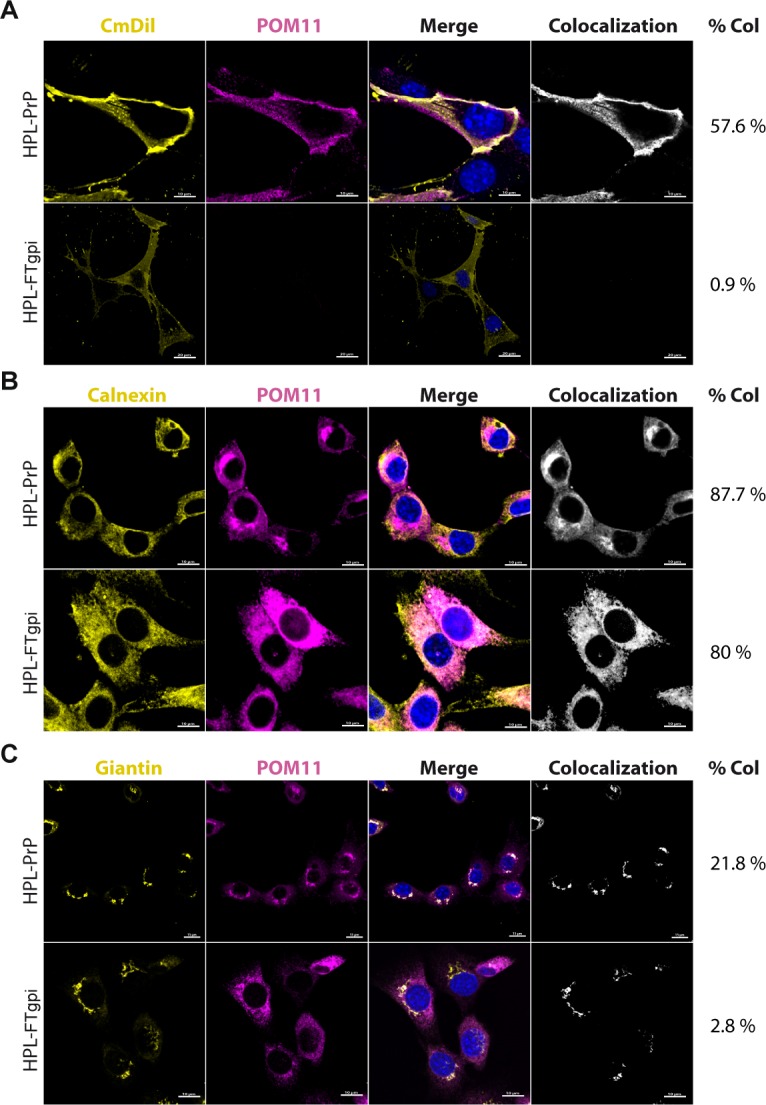
FTgpi is mostly retained in the ER. Immunofluorescence on stably transfected HPL-PrP and HPL-FTgpi cells. POM11 stains both PrP and FTgpi. (**A**) Cells were stained with the surface marker CmDil (yellow), fixed, and stained with POM11 (purple) followed by Alexa 647 anti-mouse IgG secondary antibody, and reacted with DAPI (blue) to stain the nuclei. As expected, PrP showed colocalization with the surface marker CmDil (yellow), whereas FTgpi failed to be expressed on the plasma membrane, as no signal was detected. (**B**) Cells were fixed, permeabilized and stained with calnexin antibody labeling the ER (yellow) and with POM11 (purple), followed by Alexa 647 anti-mouse and Alexa 555 anti-rabbit IgG secondary antibodies. Blue: DAPI nuclear stain. Both PrP and FTgpi showed colocalization with calnexin, confirming their presence in the ER. (**C**) Cells were fixed, permeabilized, and stained with giantin antibody labeling the Golgi apparatus (yellow), as well as POM11 (purple), followed by Alexa 647 anti-mouse and Alexa 555 anti-rabbit IgG secondary antibodies., Blue: DAPI. PrP partially but specifically colocalized with giantin, indicating that most PrP was in the ER. However, only a faint colocalization signal was detected in HPL-FTgpi. Confocal imagines were processed with Imaris; colocalization was calculated using the function ImarisColoc.

We then compared the subcellular localization of FTgpi and PrP. The surface of live HPL cells stably transfected with either full-length PrP^C^ or FTgpi was co-stained using the plasma membrane marker CmDil and POM11, an antibody against the FT of PrP^C^ [16], which recognizes both PrP^C^ and FTgpi. As expected, PrP^C^ was conspicuously stainable and colocalized with CmDil, whereas no immunofluorescent signal was detected for FTgpi (Fig. 4A), although the total FTgpi expression level was similar to that of PrP^C^ (S3 Fig.). FACS analysis on live cells confirmed that much less FTgpi was present on the plasma membrane than PrP^C^, as the FTgpi signal (red line) was slightly stronger than the background (grey line, HPL-GFP) but much weaker than PrP^C^ (blue line; S4 Fig.). This suggests that FTgpi undergoes abnormal processing, and that its routing towards the plasma membrane is impaired.

Fixed and permeabilized cells were co-stained with an antibody against calnexin, an ER marker, and POM11. Both PrP^C^ and FTgpi were found to colocalize with calnexin, confirming their presence in the ER (Fig. 4B). Conversely, co-staining with an antibody against giantin, a Golgi marker, showed colocalization with PrP^C^ as expected, but not with FTgpi (Fig. 4C). We then compared the intracellular distribution of PrP versus FTgpi in cells stained solely with POM11 (S4 Fig.). Most HPL-PrP cells showed a characteristic Golgi-like staining (S4 Fig., white arrows), which was however absent from HPL-FTgpi cells. This suggests that FTgpi was unable to leave the ER. Co-staining with LAMP2, a lysosome marker, showed minimal colocalization with PrP^C^ and FTgpi (S4 Fig.).

### FTgpi is degraded by proteasomes

**Fig 5:**
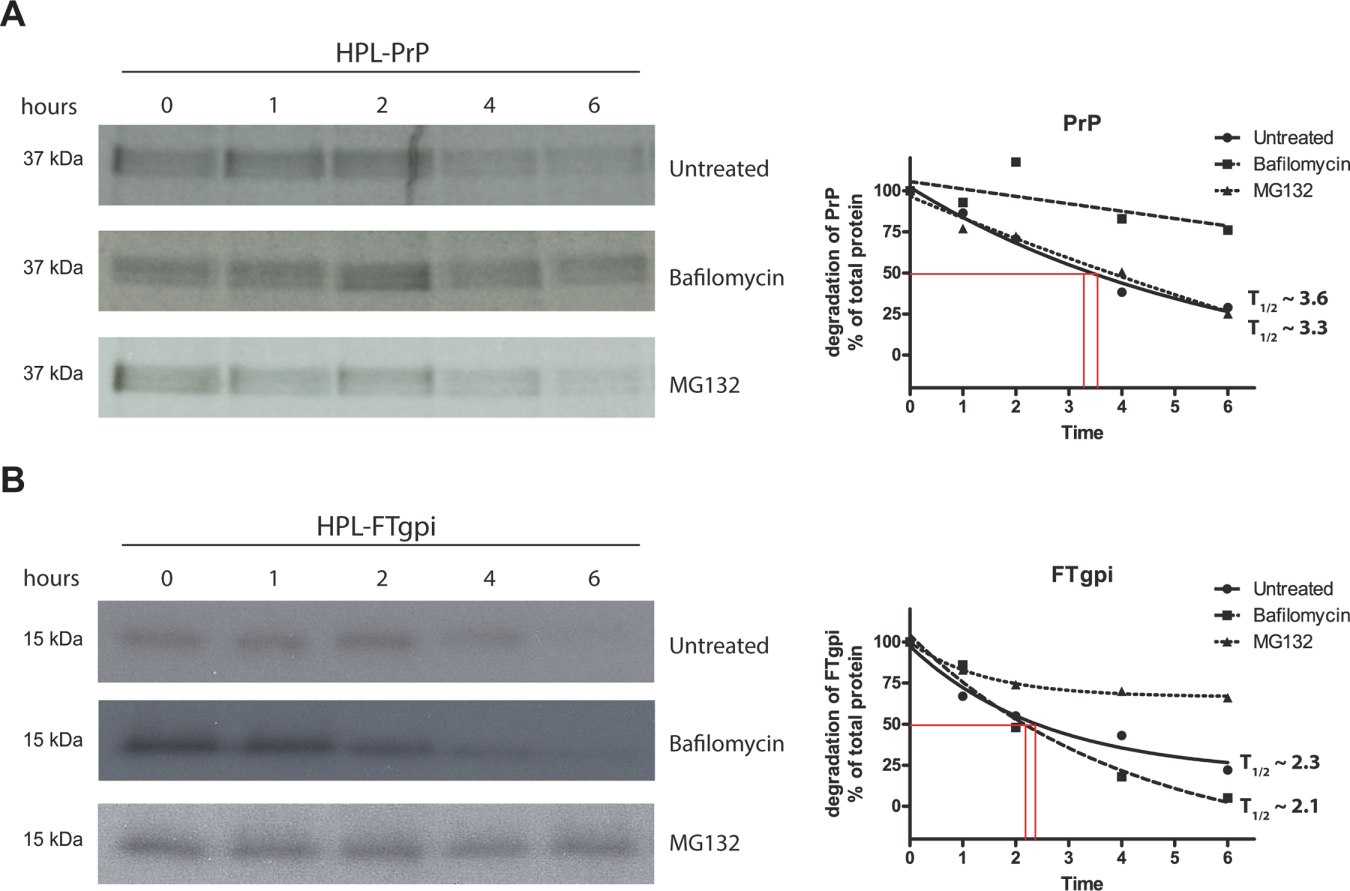
FTgpi is degraded through proteasomes. HPL cells were transiently transfected with PrP or FTgpi, and metabolically labeled with [^35^S]Met/Cys for 20 min at 37°C. Then, cells were either lysed after the pulse or incubated in culture medium without ^35^S at 37°C for 1, 2, 4, and 6 h respectively, in presence of either DMSO (untreated), bafilomycin (a lysosomes inhibitor) or MG132 (a proteasomes inhibitor). Proteins were immunoprecipitated with POM2 and subjected to SDS-PAGE and autoradiography. (**A**) Left panel: PrP turnover was impaired upon treatment with bafilomycin but not MG132. Right panel: evaluation of autoradiograms: the amounts of protein are expressed as percentage of total protein rescued directly after the labeling period and plotted as a function of the chase time points. (**B**) Left panel: FTgpi turnover was impaired upon treatment with MG132 but not bafilomycin.

The expression level of FTgpi protein in mouse brains was ~15% of that of PrP^C^ in wild-type mice despite its robust mRNA transcription, suggesting that FTgpi may be rapidly degraded. We investigated the mechanism of FTgpi clearance by pulse-chase experiments using a battery of compounds that selectively inhibited lysosomes (Bafilomycin) or proteasomes (MG132). We transiently transfected HPL cells with either FTgpi or PrP^C^, and metabolically labeled cells with [^35^S]methionine/cysteine for 1 h. Cells were either harvested directly or chased for different intervals of time in ^35^S-free culture medium before lysis. FTgpi and PrP were immunopurified with POM2, deglycosylated with PNGase F, and analyzed by SDS-PAGE. PrP^C^ resides physiologically on the plasma membrane, where it is endocytosed and recycled to the membrane [17] or, alternatively, degraded through lysosomes [18]. As anticipated, PrP^C^ turnover was significantly reduced by lysosomal inhibition such that its half-life (T_1/2_) was no longer measurable, whereas the T_1/2_ was not altered when proteasomes were inhibited (T_1/2_ ~ 3.6) compared to the untreated condition (T_1/2_ ~ 3.3; Fig. 5A). In contrast, FTgpi degradation was drastically impaired by proteasome inhibition, yet it was unaffected by lysosomal inhibition (T_1/2_ ~ 2.1 h) compared to the untreated condition (T_1/2_ ~ 2.3 h; Fig. 5B). This suggests that FTgpi is retro-translocated from the ER into the cytosol and is eventually degraded by the proteasome machinery.

### FTgpi induces ER stress *in vitro*

The fast cellular turnover of FTgpi raised the possibility that its expression may elicit ER stress and the unfolded protein response (UPR). The UPR is mediated by three families of signal transducers, ATF6, PERK and IRE1, which sense the conditions of protein folding in the ER lumen and relay the information to the cell through various mechanisms, including regulated proteolysis (ATF6), translational control (PERK), and nonconventional mRNA splicing (IRE1).

**Fig 6:**
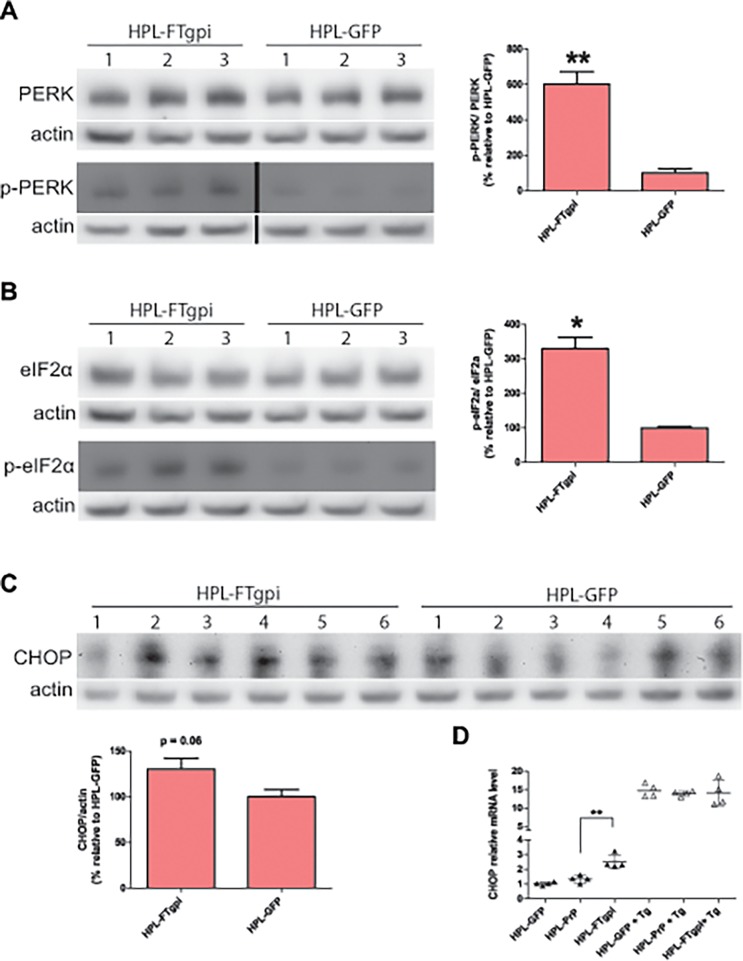
FTgpi induces ER stress in HPL cells. Cell lysates were analyzed by Western blotting. Numbers denote independent experiments. p-PERK (**A**) and p-eIF2α (**B**) were significantly higher in HPL-FTgpi than in HPL-GFP cells. p-PERK blot: non-consecutive lanes of the same blot. p-PERK/PERK and p-eIF2α/eIF2α ratios were calculated after values were normalized with actin. (**C**) CHOP was also found to be increased in HPL-FTgpi cells. (**D**) CHOP mRNA was quantified by RT-PCR. Thapsigargin (Tg)-treated cells were used as positive control. CHOP was significantly upregulated in HPL cells expressing FTgpi. (**A-B-C**) Each bar indicates the average ± SEM of 3 or 6 biological replicates. ** P<0.01 and *P<0.05 by unpaired two-tails t-test (each bar is compared to HPL-GFP). (**D**) Each sample is representative of 4 biological replicates. Error bars indicate averages ± SEM. **P<0.01 by one-way ANOVA with Bonferroni multiple comparisons post-test (each sample is compared to HPL-GFP).

We used stably transfected HPL cells to assess ER stress markers by immunoblot. HPL-FTgpi cells exhibited significantly increased phosphorylation levels of both PERK (Fig. 6A) and eIF2α (Fig. 6B) when compared with HPL-GFP. Hence, FTgpi robustly activates the PERK pathway. C/EBP-homologous protein (CHOP) expression was also found to be slightly increased (Fig. 6C), and its mRNA was significantly overexpressed (Fig. 6D). Similar results were obtained when HPL-FTgpi were compared to HPL-PrP cells (S5 Fig.).

Next, we investigated the status of the IRE1 pathway by examining specifically the spliced version of XBP1. Cells treated by Thapsigargin (Tg), which induces robust ER stress, were used as positive controls. Surprisingly, the level of spliced XBP1 in HPL-FTgpi cells was not altered compared to HPL-PrP (S6 Fig.). Secondly, we assessed the mRNA expression level of the most common markers of ER stress, whose expression is increased by the activation of ATF6 (GRP78, HERP1, EDEM1, ERP72, HSP40) [19,20]. Only HERP1 was found to be slightly upregulated (S6 Fig.). Hence IRE1and ATF6 may not be key players in the process leading to ER stress *in vitro*.

### FTgpi induces ER stress *in vivo*

**Fig 7:**
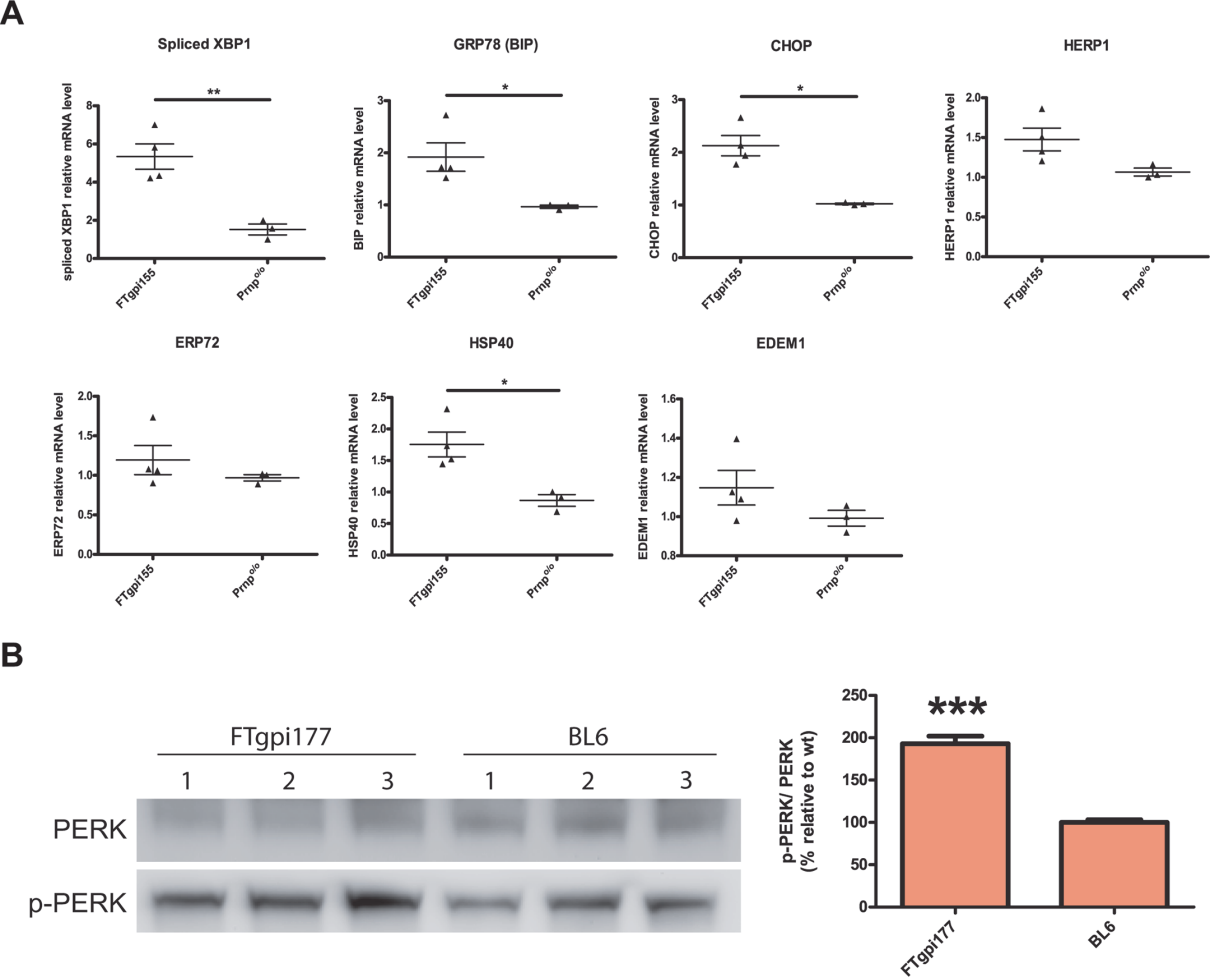
FTgpi induces ER stress *in vivo*. (**A**) mRNA was extracted from cerebella of FTgpi155 and *Prnp* ^o/o^ mice. mRNA levels of ER stress markers were quantified by RT-PCR. XBP1, BiP and CHOP were found to be significantly upregulated. *P<0.05 and **P<0.01 by unpaired two-tails t-test. (**B**) Cerebellum homogenates of FTgpi177 and *Prnp* ^o/o^ mice were analyzed by Western blot. p-PERK was increased in cerebellum of FTgpi177 mice. Blot was first decorated with p-PERK antibody, stripped for 20 min, and stained with PERK. Each bar indicates the mean ±SEM of 3 biological replicates. *** P<0.001 by unpaired two-tails t-test (each bar is compared to BL6).

In order to verify the presence of ER stress *in vivo*, we performed the same analysis using mRNA derived from the cerebellum of asymptomatic FTgpi155 *Prnp* ^o/o^ mice. mRNA levels of spliced XBP1, BiP, CHOP, and HSP40 were all significantly upregulated compared to age-matched *Prnp* ^o/o^ control mice (Fig. 7A), indicating that FTgpi causes a comprehensive ER stress response *in vivo*, with involvement of all three IRE1, PERK and ATF6 pathways. Importantly, ER stress was detected in FTgpi mice at a stage where no cerebellar degeneration was yet to be observed, suggesting that ER stress is an upstream event that may lead to the subsequent neurodegenerative process. Increased phosphorylation levels of PERK were also observed in cerebellar homogenates of FTgpi mice (Fig. 7B), supporting the evidence of ongoing ER stress *in vivo*.

### FTgpi binds BiP *in vivo*

**Fig 8:**
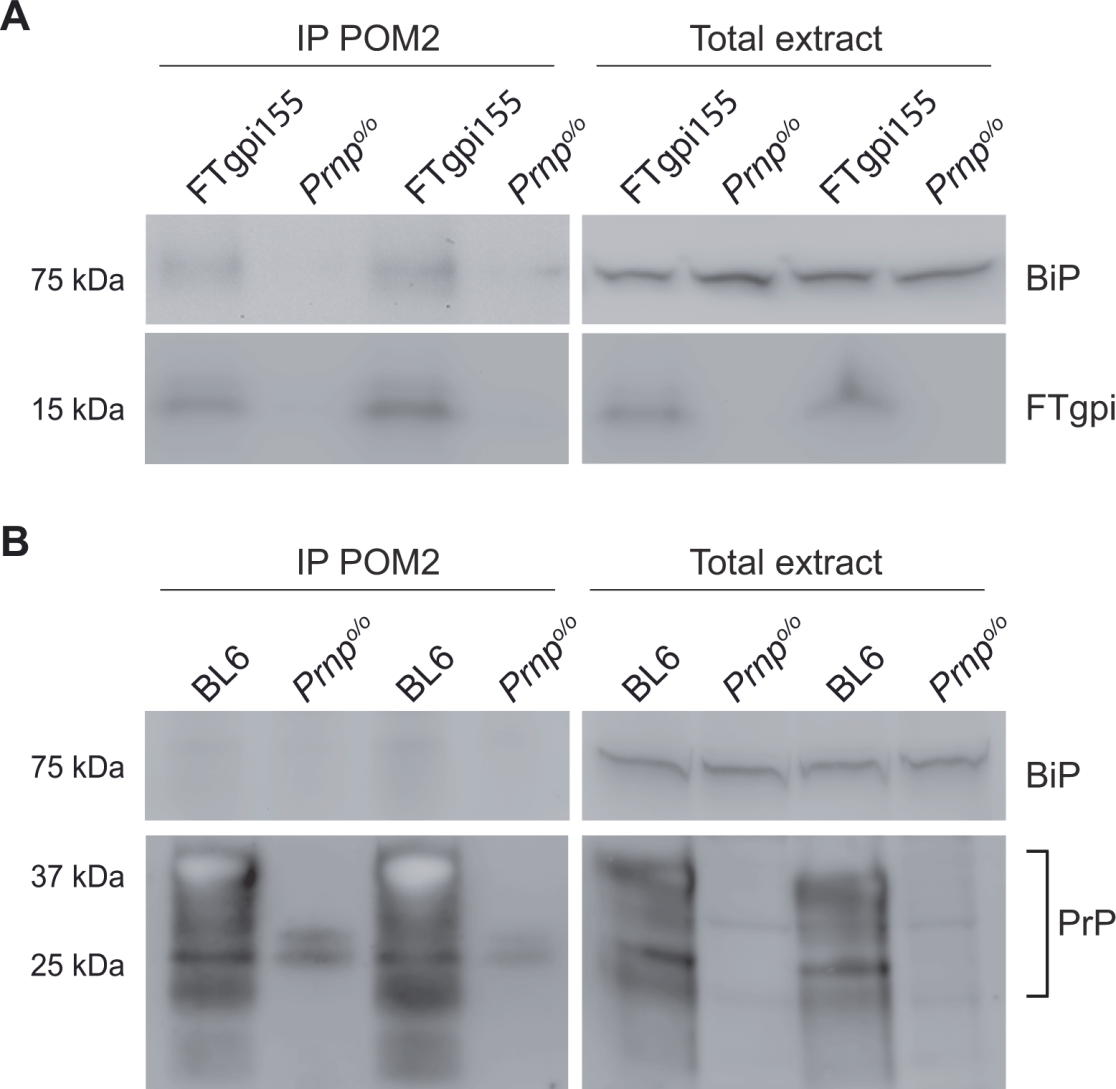
FTgpi binds BiP *in vivo*. Brain homogenates of FTgpi155, BL6 and *Prnp* ^o/o^ mice were incubated overnight with POM2 to precipitate FTgpi and PrP. Precipitates were analyzed by Western blotting (for details see Material and Methods). (**A**) Right panel: total extract. Left panel: immunoprecipitates, showing that BiP specifically co-precipitated with FTgpi, as no signal was detected in *Prnp* ^o/o^ mice. (**B**) Right panel: total extract. Left panel: immunoprecipitates, showing that BiP does not, or minimally, co-precipitated with PrP.

BiP binds to IRE1, PERK and ATF6 under normal conditions and dissociates from these UPR sensors during acute ER stress. In ER stress, chronic presence of unfolded proteins saturates the free pool of chaperones, titrating BiP away from IRE1 and thereby activating the UPR signaling [21,22]. Further, misfolded proteins in the ER that do not interact with BiP fail to induce the UPR [22]. To examine whether FTgpi interacts with BiP, we immunoprecipitated FTgpi or PrP^C^ from FTgpi155 and BL6 brains respectively using POM2. Although FTgpi protein expression is significantly lower (~15%) than that of the full length PrP^C^, we found that it specifically co-precipitated BiP (Fig. 8A), whereas BiP signal was too weak to be detected upon PrP^C^ immunoprecipitation with POM2 in BL6 brains (Fig. 8B). *Prnp* ^*o/o*^ mice were used as control (Fig. 8A-B).

## Discussion

Ligands targeting the α1 and α3 helices of the PrP^C^ globular domain induce rapid neurotoxicity [5,23]. Toxicity was prevented by deletions of the OR domain, suggesting that the FT has neurotoxic properties and is required to transmit the toxic signals originating from the globular domain [5]. But what is the nature of the deleterious effects exerted by the FT? Maybe the binding of toxic antibodies to the GD locks the FT allosterically into a restricted conformational space, leading to inappropriate interactions with membrane constituents. We reasoned that membrane anchoring, as in the FTgpi construct described here, might restrict the conformational freedom of the FT similarly to the toxic anti-GD antibodies. Indeed, mice expressing the FTgpi protein developed a severe form of ataxia and became terminally sick within few weeks after the appearance of symptoms.

Similarly to other mice with interstitial PrP deletions (collectively referred to as “ΔPrP”) [13,14,24], FTgpi mice experienced massive loss of CGNs. This predilection for cerebellar toxicity is most probably due to the high levels of transgene expression in cerebellum, which is typical for the half-genomic expression construct used here. Furthermore, cerebellar neurons are more sensitive to PrP-related pathologies than neurons in other brain regions and more recently cerebellar neurons were shown to be sensitive to ER stress compared to neurons from other regions [25].

In contrast to the ∆PrP pathologies, FTgpi phenotype was not ameliorated (and was possibly exacerbated) by co-expressing wild-type PrP^C^. This discrepancy does not necessarily prove that the mechanism of toxicity of FTgpi differs radically from that of other ΔPrP mice. Maybe the conformation of the FT is altered by deletions of the “hinge” region between FT and GD, analogously to FTgpi, but is redressed by heterooligomerization between wild-type PrP^C^ and ΔPrP [26]

### FTgpi induces chronic ER stress leading to neurodegeneration

FTgpi showed a typical ER retention phenotype with minimal exposure to the plasma membrane, cytosolic retrotranslocation, and mostly proteasomal degradation – whereas wild-type PrP^C^ is primarily mainly degraded by lysosomes. These observations raised the question whether FTgpi triggered ER stress. Indeed, FTgpi selectively activated the PERK branch of the UPR, which protects cells by transiently dampening cellular protein synthesis, thus reducing misfolded protein load and restoring ER homeostasis [7]. However, chronically activated PERK can promote cell death by downregulating the antiapoptotic BCL-2 protein [6] and by inducing CHOP, which in turn promotes the transcription of the proapoptotic protein BIM [6,27]. This signaling cascade protects the organism from rogue cells that cannot guarantee the fidelity of their signaling components [9]. Additionally, we noticed that FTgpi interacted with BiP, a key ER chaperone. This binding may reduce the availability of BiP to other essential proteins and contribute to toxicity (S8 Fig.).

The UPR can modulate the protein folding capacity of the ER by increasing the expression of chaperones via the IRE1 and ATF6 pathways [9,19,28]. The activation of these pathways was not observed in FTgpi-expressing cultured cells, but mouse cerebella exhibited a comprehensive ER stress response with increased levels of spliced XPB1, BiP, CHOP and HSP40. This discrepancy may reflect the increased capacity of immortalized cells to deal with stress, or secondary pathologies in additional cell types *in vivo*. An FT version lacking the hydrophobic core (HC) failed to induce hyperphosphorylation of eIF2α, suggesting that its hydrophobic pattern is targeted by the ER quality-control machinery [29]. In wild-type PrP^C^, the HC may be shielded by the folded globular domain. It will be exciting to explore whether the conformational transitions of PrP^C^ induced by anti-GD antibodies, and by prion infections, trigger an analogous exposure of the HC.

Prolonged UPR activity is concomitant with chronic ER stress associated cell death [8]. Importantly, ER stress was detectable in very young FTgpi mice, before any apparent histological or clinical signs of neurodegeneration. It will be interesting to investigate if pharmacological inhibition of PERK prevents neurodegeneration in FTgpi mice, although the systemic effects of such compounds prevent a long-term experiment to elucidate this phenomenon.

In some models of neurodegenerative disorders, expression of the mutant protein at levels comparable to wild-type leads to delayed onset or even to loss of the pathological phenotype. Here, however, the majority of the FTgpi protein never traverses the ER suggesting that it is prone to misfolding and that it is retained in the ER. ER retention typically leads to the activation of UPR followed by degradation of the misfolded protein. At lower expression levels, FTgpi may be expected to induce chronic ER stress owing to its continuous synthesis and accumulation in the ER, potentially with a later onset. Indeed, the late development of the symptoms in heterozygous FTgpi mice supports this interpretation.

In view of the robust transcription of FTgpi mRNA, might the neurotoxicity in FTgpi mice be nonspecifically caused by the accumulation of misfolded FTgpi in the ER? This is unlikely since FTgpi protein levels were extremely low even in terminally sick mice, suggesting that the proteasomal system was not overwhelmed and was still functional in clearing FTgpi. Proteasome disruption is thought to play a major role in many neurodegenerative disorders [30]. In the case of synucleinopathies, ER stress followed by proteasomal dysfunction results in toxic accumulation of misfolded α-synuclein [31].

### Parallels between FTgpi toxicity and transmissible spongiform encephalopathies

Prion infections are often accompanied by ER dysfunctions. Nanomolar concentrations of PrP^Sc^ from mouse brains induce apoptosis in N2A neuroblastoma cells, and toxicity is associated with increased release of calcium from the ER, strong upregulation of UPR-inducible chaperones, and higher sensitivity to ER stress-induced cell death [32,33]. Also, brain samples from cows affected by bovine spongiform encephalopathy show upregulation of genes involved in ER stress responses including ER chaperones, heat shock protein Grp94, and the chaperone Grp170 [34]. Furthermore, abnormal upregulation of protein disulfide isomerase, Grp79, and Grp58 was detected in 263K infected hamsters brain tissues [35]. Prion-infected mice show sustained UPR induction, protracted eIF2α phosphorylation and PERK activation leading to repression of translation, synaptic dysfunction and neuronal death [10], and inhibiting the PERK pathway prevented neurodegeneration [36]. These findings are strikingly similar to those in FTgpi mice. Although the molecular details of extracellular protein aggregates inducing the UPR is unknown, the downstream neurotoxicity in both cases follows the activation of UPR and follows activation of identical cascades. Thus FTgpi could function as an excellent model system to understand the toxicity mediated events downstream of activation of UPR. It will also be very interesting in a future study to address the self-aggregation and transmission capabilities of FTgpi, which can shed light on the role of FT as an inducer of prion toxicity.

### Relevance of FTgpi toxicity beyond prion diseases

Alterations in p-PERK, p-eIF2α and CHOP are seen in patients with Parkinson’s (PD) [31,37] and Alzheimer’s diseases (AD) [38–40], Huntington’s disease (HD) [41,42], and ALS [28,43]. ER dysfunctions may therefore represent a common pathway for many neurological diseases [44]. In particular, Aβ toxicity has been claimed to be mediated by PrP^C^ and Aβ oligomers have a high affinity for the FT [45]. Although these findings are controversial and may not be universally valid [46], they suggest that FT-mediated toxicity may be at work also in non-prion-related neurodegeneration.

## Material and Methods

### Ethics statement

Mice were bred in high hygienic grade facilities and housed in groups of 3–5, under a 12 h light/12 h dark cycle (from 7 am to 7 pm) at 21±1°C, with sterilized food (Kliba No. 3431, Provimi Kliba, Kaiseraugst, Switzerland) and water *ad libitum*. Animal care and experimental protocols were in accordance with the Swiss Animal Protection Law, and approved by the Veterinary office of the Canton of Zurich (permits 130/2008 and 41/2012).

### Construction of transgenes

To create the FTgpi construct, total mouse brain cDNA was amplified using the primer set SY6 and SY7 which introduce *Bam*HI and *Sal*I cloning sites at the 3’ and 5’ ends of the ORF of PrP. PrP was cloned into the cloning vector pBSK (pBSK-PrP). This plasmid served as a template for the following cloning steps. An *Xba*I cloning site was introduced by PCR in the PrP ORF just before amino acid 225. The primer sets used were SY6/SY6a and SY7a/SY7. An amino-terminal insert was amplified from pBSK-PrP using the primer set SY6/SY8 which introduces *Bam*HI and *Xba*I cloning sites before the start codon and after amino acid 140, respectively. pBSK-PrP and the amino-terminal insert were digested with *Bam*HI and *Xba*I, and the open vector and the insert were ligated, to generate a construct consisting of the first 140 amino acids of PrP directly linked amino acid 226 (PrP_Δ141–225_). Primers: SY6 fw (5’-CGC GGA TCC AAT TTA GGA GAG CCA AGC AGA-3’), SY6a rev (5’-CGC TCT AGA ACG TCG CCC GTC GTA ATA GGC-3’), SY7a fw (5’-CGC TCT AGA GAC GGG AGA AGA TCC AGC AGC-3’), SY7 rev (5’-ACG CGT CGA CCA CGA GAA TGC GAA GGA ACA-3’), SY8 rev (5’-ACG CTC TAG ACC AGT CGT TGC CAA AAT G-3’).

### Generation of transgenic mice

The phg plasmids containing the PrP_Δ141–225_ (FTgpi) ORF and PrP_Δ112–254_ (sFT) were propagated in Escherichia coli XL1 blue, the minigene were excised with *Not*I and *Sal*I, processed as described [11], and injected into fertilized *Prnp* ^*+/+*^ oocytes (B6D2F1/Crl) by standard procedures [47]. The transgene positive founders were crossed with *Prnp* ^o/o^ ZHI mice in order to obtain transgene-positive mice on a *Prnp* ^o/o^ background. FTgpi transgene was identified by PCR using the exon 2 forward primer pE2 and the non-coding region at 3’ of exon 3 reverse primer 3’NC. The fragment size of the transgene was 647bp. The sFT_ΔHC_ transgene was identified by PCR using the forward primer pE2 and the reverse primer sFT3’. The fragment size of the transgene was 417 bp. In order to outbreed the *Prnp* ^*+*^ allele, PCR analysis was carried out using primers P10 (forward primer, *Prnp* exon 3), 3'NC (reverse primer), and P3 (reverse primer, neoR gene); P10 and 3'NC gave a 542 bp signal for the *Prnp* ^*+*^ allele and P3 and 3'NC gave a 362 bp product for the *Prnp* ^*0*^ allele. In the PCR, the transgene was also detected as a 269 bp product. Primers: pE2 (5’-CAA CCG AGC TGA AGC ATT CTG CCT-3’), 3’NC (5'-CCC TCC CCC AGC CTA GAC CAC GA-3’), P10 (5'-GTA CCC ATA ATC AGT GGA ACA AGC CCA GC-3’), P3 (5'-ATT CGC AGC GCA TCG CCT TCT ATC GCC-3’), sFT3’ rev (5’-CCT ATC TCA CAC ATG CTT GAG GTT GGT TTT TGG −3’).

### Morphological analysis

Brains were removed and fixed in 4% formaldehyde in PBS, pH 7.5, paraffin embedded, and cut into 2–4 μM sections. Sections were stained with hematoxylin and eosin (H&E) and commercial antibodies to GFAP (glial fibrillary acidic protein; activated astrocytes). The same protocol was applied to livers, spleens, kidneys, hearts, stomachs, pancreas and intestines.

### TUNEL staining

5 μM frozen sections were fixed with 1% paraformaldehyde in PBS, pH 7.4, for 10 min at room temperature (RT), rinsed twice in PBS, permeabilized with a solution of Ethanol:Acetic acid (2:1) for 5 min at −20°C, rinsed twice with PBS, and then stained using ApopTag Plus *In Situ* Apoptosis Fluorescein Detection kit, according to the manufacturer’s directions (Millipore). Cell nuclei were counterstained with DAPI. Sections were mounted with Fluorescent Mounting Medium (Dako) and imaged on a CLSM Leica SP5 ZMB. Co-staining TUNEL and Iba1 on frozen sections: 5 μM slices were cut and put on glass slides, fixed 10 min in 1% paraformaldehyde in PBS, washed 2X with PBS, 2 min in 50% acetone, 2 min in 100% acetone, 2 min in 50% acetone, washed 2X with PBS and 1X with PBS-T. Slides were treated 10 min with Protein block serum free (Dako), incubated 2h at RT with Iba1 diluted in 0.3% Triton in PBS, washed 2X with PBS, 1X with PBS-T, incubated with anti-rabbit Alexa467 secondary antibodies diluted in 0.3% Triton in PBS 1h at RT with 1 ug/ml DAPI. Slides were washed 2X with PBS, 1X with PBS-T, and stained using ApopTag Plus In Situ Apoptosis Fluorescein Detection kit, according to the manufacturer’s directions (Millipore). Sections were mounted with Fluorescent Mounting Medium (Dako) and imaged on a Olympus Fluoview FV10i.

### Mice survival study

Survival of the mice bearing the FTgpi transgene were monitored once a week for clinical signs, which might include one or more of the following: weight loss, hunch posture, rough hair coat, limb parexis, ataxia. The mice were scored based on the clinical symptoms and once they were assigned as grade 2, they were observed more frequently and were euthanized humanely at the end point (which was when the mice reached grade 3.5 for more than 4 days) using carbondioxide inhalation. For more details of how the clinical signs were scored please see the Clinical scoring table (S2 Table).

### Immunofluorescence

Cells were grown on Cultureslides, 8-well (BD Biosciences), washed 1X PBS and fixed with 4% formalin in PBS, pH 7.4, for 15 min at RT. After washing 2X with ice-cold PBS, cells were permeabilized with 0.3% Triton X-100 in PBS for 5 min at RT, rinsed 1x with PBS, and incubated for 40 min in blocking solution (1% FBS in PBS) for 40 min at RT. Cells were rinsed 1x with PBS and incubated with primary antibody (POM11, giantin, LAMP2) diluted in washing buffer (1%BSA, 0.25% Triton X-100 in PBS) overnight at 4°C. After washing 2X with PBS, cells were incubated with Alexa Fluor-conjugated 555- anti-mouse or 647-secondary anti-rabbit or anti-rat antibodies (Invitrogen, Molecular Probes) diluted 1:5000 in washing buffer for 2h at RT, and incubated with 1 ug/ml DAPI (4’,6-diamidino-2-phenylindole, Invitrogen). Permeabilization with 100% methanol at −20°C for 10 min was performed when calnexin was used as a primary antibody. Surface staining with CmDil: CM-Dil was diluted in cell culture media (OPTI-MEM, 10% FBS, Glutamax) at a concentration of 0.5 ug/ml, immediately before labelling. Cells were incubated at 37°C for 5 min, then at 4°C for 15 min, washed 1X with PBS and fixed in 4% formalin. Primary and secondary antibodies stainings were performed as previously described. Cells were mounted with Fluorescent Mounting Medium (Dako) and imaged on a CLSM Leica SP5 ZMB. Confocal imagines were processed with Imaris; colocalization was quantified using the function ImarisColoc after setting the thresholds.

### Western blot analysis

Cerebellums were homogenized using TissueLyser LT for 5 min in 10 vol of lysis buffer (0.5% Nonidet P-40, 0.5% 3-[(3-cholamidopropyl)dimethylammonio]-1-propanesulfonate (CHAPS), protease inhibitors (complete Mini, Roche), phosphates inhibitors (PhosphoSTOP, Roche) in PBS, and centrifuged at 1000g for 5 min at 4°C to remove debris prior to analysis by SDS-PAGE (Novex NuPAGE 10% Bis-Tris Gels). After electrophoresis, samples were transferred to nitrocellulose membranes (PROTRAN, Whatman). Membranes were blocked with 5% milk 1h at RT and incubated overnight in Tris-buffered saline-Tween (TBS-T) at 4°C with primary antibodies, followed by incubation with a secondary Peroxidase-Goat Anti-Mouse IgG (H+L) (#62–6520) or Peroxidase-Goat Anti-Rabbit IgG (H+L) (#111.035.045), used at 1:10000 1h at RT. Membranes were developed with Luminata Crescendo (Millipore) and images were acquired using Stella imaging system (Raytest). Cells were homogenized on ice using a 30G syringe in lysis buffer, centrifuged at 1000g for 5 min at 4°C to remove debris prior to analysis by SDS-PAGE (E-PAGE 48 wells 8%). After electrophoresis, samples were transferred using the iBlot system (Invitrogen). For deglycosylation, denatured total protein was incubated at 37°C for 4 h with 500 U PNGase F (New England Biolabs) according to the manufacturer’s instructions.

To identify if the FTgpi was indeed gpi anchored, lysate from the mouse brain (500ug) was resuspended in ice cold PBS at a final concentration of 4mg/ml. 2% precondensed Triton X-114 was added to the suspension so as to make it 1/5 the volume. This was followed by incubation on ice for 15 min with occasional stirring and followed by centrifugation for 10 min at 10000g. The cold supernatant fraction was further processed by warming it up in the water bath at 37°C until it is cloudy followed by centrifugation at 1000g for 10 min. The upper phase is detergent depleted phase and contains the soluble proteins whereas the lower detergent enriched phase contains the GPI anchored proteins.

For the PI-PLC digestion, 500ug of lysate from HPL cells transfected with FTgpi for 24h was diluted in a PI-PLC dilution buffer (50 mM Tris⋅HCl, pH 8.0, 5 mM EDTA, 1% NP-40) and PI-PLC (Sigma) was added at a concentration of 1.5U/ml overnight on a spinning wheel at room temperature with a final reaction volume of 100ul. After the reaction 50ul was directly used for migration of SDS PAGE to identify both the gpi anchored and the cleaved fragments. The detection was done using POM2 antibody.

### Immunoprecipitation

Protein extraction from mouse cerebella was performed using mechanical lysis in IP buffer (HBS buffer (pH 6.8) with 2% CHAPS and cocktail of protease inhibitors (Roche)). This was followed by centrifugation at 8000 rpm for 10 min. The amount of protein in the lysate was estimated using BCA assay and 500 μg of the protein was used for the immunoprecipitation assays. For the cell lines, lysis was performed for 20 min at 4°C in IP buffer followed by centrifugation for 10 min at 10000 g. In both cases supernatants were precleared and incubated for 16 h at 4°C with antibodies and dynabeads. For immunoprecipitation of BiP 2 U/ml of Apyrase was added to the lysate to deplete ATP. After immunoprecipitation, the beads were washed for three times with the IP buffer and resuspended in the sample buffer (2x) after the final wash. The samples were heated at 95°C for 5 min and migrated on 12% Tris-Bis gels with the MOPS buffer.

### Pulse-chase assays

To monitor the half-life of FTgpi and PrP^C^, HPL cells were transfected with mammalian expression plasmids of the two proteins. 24 h post transfection, the cells were either treated with DMSO, MG132 (10μM) or bafilomycin (250 nM) for 2 h followed by incubation in starvation medium (DMEM without methionine and cysteine) for 40 min along with the drugs to deplete the endogenous stores of methionine and cysteine. The cells were then labeled with 50μCi/ml ^35^S-methionine/cysteine for 20 min followed by a chase in normal medium at different time points. After the chase, the cells were harvested an isotonic HEPES buffer (pH-6.8) containing 2%CHAPS and protease inhibitor cocktail. Post nuclear supernatants were obtained by centrifuging the sample at 10000g for 10 min. FTgpi and PrP^C^ were immunoisolated from the post nuclear supernatant by overnight incubation with POM2 antibody followed by incubation with Dynabeads for 2h at 4°C. The immunoprecipitates were migrated on a 12% Tris-BIS gels followed by fixation and drying of the gels. The dried gels were exposed to phosphoscreen and the radiolabelled products were revealed using a film.

### FACS analysis

Cells were harvested with EDTA 10 mM, washed 2X in PBS, resuspended in ice-cold FACS buffer (2%FBS, 2mM EDTA in PBS) and incubated with 1:500 POM11 antibody for 30 min on ice. Cells were then washed 2X with ice-cold FACS buffer and incubated for 30 min on ice with Alexa Fluor-conjugated 647 anti-mouse, protected from light. Cells were washed 3X with ice-cold FACS buffer and transferred into micro-FACS tubes. Data was acquired using FACSCalibur (BD Biosciences).

### Antibodies

Mouse monoclonal anti-PrP antibodies POM1, POM2, POM11 [16] were diluted 1:5,000 for Western blot (0.5 μg/ml); anti-PERK (#3192), anti-p-PERK (#3179), anti-eIF2α (#9722), anti-p-eIF2α (#9721), anti-CHOP (#5554), anti-AKT (#4685), anti-p-AKT (#4060), (Cell Signaling) were diluted 1:1000 for Western Blot; actin antibody (Millipore AG, MAB1501R) was used at 1:10000 for Western blot. Immunofluorescence: anti-calnexin (Abcam, ab22595) was used at 4 ug/ml, anti-giantin (Abcam, ab24586) was used at 1/1000, anti-lamp2 (Abcam, ab25339) was used at 1/100, anti-Iba1 (Abcam, ab5076) was used at 1/100, and POM11 was used at 1/1000. Co-staining TUNEL and Iba1: anti-Iba1 (Wako, 019–19741) was used at 1:500.

### mRNA analysis

Cerebellar RNA was isolated using RNeasy Plus Universal Mini Kit (QIagen), according to the manufacturer’s manual. After reverse transcription (QuantiTect Rev. Transcription Kit, QIagen), cDNA was used for RT-PCR to quantify FTgpi and PrP mRNA levels using the following primers: pE2 fw (5’-CAA CCG AGC TGA AGC ATT CTG CCT-3’), sFT ORF rev (5’-AGG TGC CAC CCT GAG GTG GGT AA-3’). GAPDH was used to standardize expression levels using primers GAPDH fw (5’-CCA CCC CAG CAA GGA GAC T-3’) and GAPDH rev (5’-GAA ATT GTG AGG GAG ATG CT-3’). RT-PCR was performed using SYBR-green (Roche) and determination of ΔΔCT-values was done on a ViiA 7 real-time system (Applied Biosystems). Cell mRNA was isolated using RNeasy Mini Kit (QIagen), according to the manufacturer’s manual. After reverse transcription (QuantiTect Rev. Transcription Kit, QIagen), cDNA was used for RT-PCR to quantify the ER stress related genes; CHOP: fw (5’-CTT CAC TAC TCT TGA CCC TGC-3’), rev (5’-CTA GTT CTT CCT TGC TCT TCC TC-3’). Spliced-Xbp1 primers: fw (5’-TGC TGA GTC CGC AGC AGG TG-3’), rev (5’-CAT GAC AGG GTC CAA CTT GTC-3’). BiP primers: fw (5’-CGA GGA GGA GGA CAA GAA GG-3’), rev (5’-CAC CTT GAA TGG CAA GAA CT-3’). HERP 1 primers: fw (5’-CAT CTC TAG GCC TGA GGC TG-3’), rev (5’-GTT CCT GAT GCA GCA GTG GCA-3’). ERP72 primers: fw (5’-TGG ACA CCT CCA CCT GAA GTC-3’), rev (5’-CTC ATA CTC AGG GGC AAG TTT C-3’). HSP40 primers: fw (5’-GAT CTG CCA CTG CTT CTC TAA G-3’), rev (5’-CCT GAG CAG CTT CAT AAT CC-3’). EDEM1 primers: fw (5’-GTT CTG ATA GGG GAT GTG GAA G-3’), rev (5’-GAG GAG ATA TGT GGA CTC CAC-3’). GAPDH was used to standardize expression levels. RT-PCR was performed using SYBR-green (Roche) and determination of ΔΔCT-values was done on a ViiA 7 real-time system (Applied Biosystems).

## Supporting Information

S1 Fig Models of PrP-mediated toxicity. (**A**)The cellular prion protein PrP^C^ consists of a long flexible tail (FT) and a globular domain (GD) tethered to the membrane (blue) via a GPI anchor (green). (**B**) GD ligands (e.g. antibodies and derivatives thereof) trigger neuronal death, yet this process does not occur if the FT is shortened or is sterically engaged by antibodies (not shown). This suggests that the FT is the executor of toxicity, and forms the basis of the present study. (**C**) We now show that expression of a membrane-tethered version of the FT suffices to trigger toxicity. The proximate trigger of antibody-induced toxicity may be the forced juxtaposition of the FT to the membrane. (**D**) It is tantalizing to speculate that similar mechanisms may be operative during bona fide prion disease. Infectious PrP^Sc^ oligomers may interact with PrP^C^ and create perturbations of the FT as in panels B and C.
(EPS)

S2 Fig H&E staining of FTgpi organs (A) No abnormalities were reported in liver, spleen, kidney, heart, stomach, pancreas and intestine. (**B**) Western blotting using POM11 showed FTgpi expression exclusively in the brain. Unspecific bands are present in both FTgpi and BL6 blots.
(EPS)

S3 Fig (A) Stably transfected HPL cells lysates were subjected to PNGase F treatment as indicated and analyzed by Western blotting using POM11. FTgpi does not have glycosylation sites. (B) 500ug of the protein extracted from BL6 and FTgpi 155 mice was subjected to the Triton X114 phase separation and the upper indicates the detergent depleted fraction and whereas the lower indicated the detergent rich fraction containing the gpi anchored proteins. **(C)** PI-PLC digestion (1.5U/ml) was performed overnight on 500ug of the protein isolated from HPL cells which were transfected with FTgpi for 24h and 50ul of the sample was directly loaded on the SDS PAGE to monitor both the gpi anchored and the cleaved fraction.
(EPS)

S4 Fig A minor fraction of FTgpi reaches the plasma membrane. (**A**) FACS analysis: HPL-GFP (grey line, background fluorescence), HPL-FTgpi (red line) and HPL-PrP (blue line, positive control) cells were stained with POM11, followed by Alexa 647 anti-mouse IgG secondary antibody. Each histogram is representative of three biological replicates (left panel). HPL-FTgpi median fluorescence intensity (MFI) was dramatically lower than that of HPL-PrP (right panel), indicating its scarce expression on the plasma membrane. ****P<0.0001 by unpaired two tails t-test. (**B**) Cells were fixed and stained with POM11 (purple), followed by Alexa 647 anti-mouse IgG secondary antibody, and reacted with DAPI (blue) to stain the nuclei. White arrows point at the characteristic Golgi-staining, which is widely present in HPL-PrP cells, whereas HPL-FTgpi cell do not show such signal. HPL-GFP were transfected with the pBMN empty-vector and used as control for unspecific signals. (**C**) Cells were fixed and stained with LAMP2 antibody to mark the lysosomes (yellow) and POM11 (purple), followed by Alexa 647 anti-mouse and Alexa 555 anti-rabbit IgG secondary antibodies, and reacted with DAPI (blue) to stain the nuclei. Neither PrP nor FTgpi showed colocalization with LAMP2.
(EPS)

S5 Fig FTgpi, but not PrP, triggers ER stress in HPL cells. Cell lysates were analyzed by Western Blotting using the indicated antibodies. Numbers: biological replicates. (**A**) p-PERK and p-eIF2α were significantly increased in HPL-FTgpi cells. Values were normalized with actin. Each bar indicates the mean ±SEM of 3 biological replicates. *P<0.05 by unpaired two-tails t-test (each bar is compared to HPL-PrP).
(EPS)

S6 Fig Transcriptional analysis of additional ER stress markers (A) Tg: Thapsigargin. Cells were treated with 0.5 μM Tg for 3 h at 37°C in order to induce ER stress as positive controls for our primers. mRNA levels were quantified by RT-PCR. Major players involved in the IRE1 and ATF6 pathways, such as spliced XBP1 and BiP respectively, were unaltered *in vitro*. Black triangles: non-treated cells; white triangles: Tg-treated cells. *P<0.05 by one-way ANOVA with Bonferroni multiple comparisons post-test.
(EPS)

S7 Fig AKT phosphorylation *in vitro* and *in vivo*. (**A**) Cell lysates of HPL-PrP and HPL-FTgpi cells were analyzed by Western blot. p-AKT was significantly decreased in HPL-FTgpi cells. p-AKT/AKT ratio was calculated after values were normalized with actin. Numbers: biological replicates. (**B**) Cerebellum homogenates of FTgpi177 and *Prnp* ^o/o^ mice were analyzed by Western blot. p-AKT was increased in cerebellum of FTgpi177 mice. Blot was first decorated with p-AKT antibody, stripped for 20 min, and stained with AKT. (**A-B**) Each bar indicates the mean ±SEM of 3 biological replicates. *** P<0.001 by unpaired two-tails t-test (each bar is compared to HPL-PrP (**A**) or *Prnp* ^o/o^(**B**)).
(EPS)

S8 Fig Schematics of the hypothetical mechanism of toxicity in FTgpi transgenic mice. FTgpi binds BIP *in vivo*, which may reduce the availability of BIP to other essential proteins, slowly inducing sustained UPR, which in turn causes cumulative ER stress through the activation of mainly PERK and XBP1 branches. Chronic activation of these pathway leads to CHOP overexpression and eventually results in synaptotoxicity and cell death.
(EPS)

S1 Table FTgpi is inherited as an autosomal trait at Mendelian frequency. (PDF)

S2 Table Clinical scoring and observation. (PDF)

S3 Table ARRIVE guidelines checklist. (PDF)

S1 Video FTgpi-induced symptoms *in vivo*. FTgpi155 mouse shows shivering, hind limb weakness and ataxia.
(M4V)

S2 Video FTgpi-induced symptoms *in vivo*: cage grid test. FTgpi155 mouse is placed on a metal grid. It cannot grasp the bars firmly, and both legs consistently fall through the grid.
(M4V)
