# Supplementary material for: Neurodegeneration and Unfolded-Protein Response in Mice Expressing a Membrane-Tethered Flexible Tail of PrP
Source: PLoS One. 2015 Feb 6;10(2):e0117412. doi: 10.1371/journal.pone.0117412 (PMC4319788; doi:10.1371/journal.pone.0117412)
Supplement: S1 Table — (PDF) [file pone.0117412.s009.pdf]

FTgpi is inherited as an autosomal trait at Mendelian frequency

Chi-square test (ratio 1:1)	Observed value	Expected value	χ^2
FTgpi +	14	13	0.08
FTgpi -	12	13	0.08
Total	26	26	0.15

Degrees of Freedom: 1. H_0: FTgpi+ = FTgpi- $\chi^2_{\mathbf{0.15}}$ p=0.7 > $\chi^2_{3.84}$ p =0.05 The observed χ^2 is not significantly different from the expected value, confirming the null hypothesis. The observed numbers are consistent with those expected under Mendel's law.
