# Supplementary material for: Neurodegeneration and Unfolded-Protein Response in Mice Expressing a Membrane-Tethered Flexible Tail of PrP
Source: PLoS One. 2015 Feb 6;10(2):e0117412. doi: 10.1371/journal.pone.0117412 (PMC4319788; doi:10.1371/journal.pone.0117412)
Supplement: S2 Table — (PDF) [file pone.0117412.s010.pdf]

Clinical scoring and observation

For clinical assessment: the mice will be observed once a week after weaning for clinical signs which might include one or more of the following: weight loss, hunched posture, rough hair coat, limb paresis, ataxia, or decreased activity. Once the mice have been scored grade 2 they will be observed more frequently as described below. Mice will be euthanized if they have grade 3.5 and more for > 4 days, as described below.

Score	clinic	assessment	action
0.00	no detectable signs of abnormal movement		
1.00	limp tail and hind limb weakness	(occasional grid test positive)	provide food in the cage
2.00	unilateral partial hind limb paralysis	one leg consistently falls through the grid (cage test)	provide food and water in the cage observe twice a week
2.50	bilateral partial hind limb paralysis	cage grit test bilateral	observe every second day
3.00	complete bilateral hind limb paralysis		observe daily; sacrifice if persistent > 7 days
3.50	complete bilateral hind limb paralysis and partial forelimb paralysis		sacrifice if persistent > 4 days
4.00	moribund (mouse completely paralyzed)		sacrifice immediately
5.00	dead		

-
